# Understanding how people with Parkinson's disease turn in gait from a real-world in-home dataset

**DOI:** 10.1016/j.parkreldis.2022.11.007

**Published:** 2022-12

**Authors:** Catherine Morgan, Jack Jameson, Ian Craddock, Emma L. Tonkin, George Oikonomou, Hanna Kristiina Isotalus, Farnoosh Heidarivincheh, Ryan McConville, Gregory J.L. Tourte, Kirsi M. Kinnunen, Alan Whone

**Affiliations:** a Translational Health Sciences, University of Bristol, 5 Tyndall Ave, Bristol, BS8 1UD, UK; b Movement Disorders Group, Bristol Brain Centre, North Bristol NHS Trust, Southmead Hospital, Southmead Road, Bristol, BS10 5NB, UK; c Faculty of Engineering, University of Bristol, Digital Health Offices, 1 Cathedral Square, Bristol, BS1 5DD, UK; d Research and Development, IXICO, 4th Floor, Griffin Court, 15 Long Ln, Barbican, London, EC1A 9PN, UK

**Keywords:** Remote sensing technology, Home environment, Gait analysis, Mobility, Parkinson's disease

## Abstract

**Introduction:**

Turning in gait digital parameters may be useful in measuring disease progression in Parkinson's disease (PD), however challenges remain over algorithm validation in real-world settings. The influence of clinician observation on turning outcomes is poorly understood. Our objective is to describe a unique in-home video dataset and explore the use of turning parameters as biomarkers in PD.

**Methods:**

11 participants with PD, 11 control participants stayed in a home-like setting living freely for 5 days (with two sessions of clinical assessment), during which high-resolution video was captured. Clinicians watched the videos, identified turns and documented turning parameters.

**Results:**

From 85 hours of video 3869 turns were evaluated, averaging at 22.7 turns per hour per person. 6 participants had significantly different numbers of turning steps and/or turn duration between “ON” and “OFF” medication states. Positive Spearman correlations were seen between the Movement Disorders Society-sponsored revision of the Unified Parkinson's Disease Rating Scale III score with a) number of turning steps (rho = 0.893, p < 0.001), and b) duration of turn (rho = 0.744, p = 0.009) “OFF” medications. A positive correlation was seen “ON” medications between number of turning steps and clinical rating scale score (rho = 0.618, p = 0.048). Both cohorts took more steps and shorter durations of turn during observed clinical assessments than when free-living.

**Conclusion:**

This study shows proof of concept that real-world free-living turn duration and number of turning steps recorded can distinguish between PD medication states and correlate with gold-standard clinical rating scale scores. It illustrates a methodology for ecological validation of real-world digital outcomes.

## Highlights

• People with PD turn (in gait) on average 22.7 times per hour during waking hours at home.
• Turning parameters can differentiate between “ON” and “OFF” medication status in PD.
• Real world turning parameters correlate with gold-standard clinical rating scales.
• In-home turns look very different when the person is observed by a clinician.
• Wall-mounted cameras with human annotators can be used to validate sensor data.

Gait and turning abnormalities are common features of PD with over half of patients reporting difficulties with turning [1] – when someone moves round on their axis while upright, changing the direction they face. Turning changes associated with PD include the ‘en bloc’ phenomenon where upper and lower body segments turn simultaneously [2,3], a longer duration of turn, less accurate turn completion, a narrower base of support [4] and the use of ‘step turns’ rather than ‘pivot turns’ [2]. Mobility outcomes in PD, can vary in nature and severity across the day and can be influenced by medication intake [5], and extrinsic factors such as stress [6].

Mobility dysfunction (of which turning is a component) can significantly reduce quality of life [7]. Understanding turning is also particularly important since turning abnormalities can predispose to falls, therefore turning parameters could potentially be used as measures predicting the time to falls in a patient with PD [1].

In unmedicated early-stage PD, gait parameters from turning are more sensitive to change compared to straight-ahead gait outcomes [8] and show promise as markers of disease progression [9], making measuring aspects of turns potentially of specific use in clinical trials of disease-modifying interventions which typically recruit recently diagnosed patients [10].

The current gold-standard clinical rating scale used in clinical trials to measure mobility outcomes, the Movement Disorders Society-sponsored revision of the Unified Parkinson's Disease Rating Scale motor sub-score (MDS-UPDRS III) [11] has limitations including related to its ‘snapshot’ nature which cannot capture real-world functional turning performance of patients [12], its non-linear and discontinuous scoring system, the inter-rater variability [13] and Hawthorne effect [14] of being observed on how someone mobilizes [15,16].

To overcome this, much work has been put into developing technological sensors, particularly wearable devices, which can detect [17] and quantify [[18], [19], [20], [21], [22]] aspects of how someone turns in gait, with some of this work done in home settings. However, even though wearable algorithms evaluating gait have been shown to poorly translate from lab to home [9] and we know that people mobilise differently between laboratory and home settings [20,21], the challenge of ‘ecological validation’ (whether the findings of the research can be applied to real world situations) remains: without a clinician present, or video cameras recording in-home, the “ground truth” of what is actually happening when the sensor predicts a turn, is missing. Attempts at ground truth have been made using clinician-held video cameras in laboratory [23] and home [24], but this does not overcome the problem that gait performance in PD can change purely due to being observed by a clinician/researcher [16]. Work has been done showing acceptability of using such high-resolution video recording for validation purposes in home settings in PD [25,26].

Other unmet needs in this space include: identifying which of the many gait parameters are most useful as surrogate markers of overall mobility in PD, and for which patient sub-groups within PD [21]; understanding what the parameters tell us (e.g. rates of progression and medication status); and more fully understanding the impact of clinician observation and/or setting on gait in PD. Additionally, there has been little work looking at turning in the practically defined “OFF” versus “ON” medication state in PD, which is particularly relevant to neuroprotective/neurorestorative trials.

The novelty of this work is in the collection of unobtrusive video data providing ground truth from a home-like setting, where most of the participant data is with no researcher present. Secondly, this dataset has been watched and annotated (where turns are detected and aspects quantified) post-hoc by clinician raters manually, providing a rich resource with which to understand real-world turning behavior. Thirdly, this data affords the comparison between human-observed (structured) and unobserved (unstructured) turning, from the same home-like setting. Controlling for the setting could give specific insight into the impact of clinician observations on gait.

We hypothesized that real-world in-home turning parameters would show promise in discriminating between PD and control participants and between the “ON” and “OFF” medication state in PD. Furthermore, we hypothesized that turning parameters would have face validity in free-living, shown by correlations with the current gold-standard clinical assessments. A sub-objective was to explore the difference in how someone turns during a clinical assessment compared to living freely. The long-term goal is to advance digital measures of mobility which could serve as critical markers of disease progression for use in clinical trials; and secondly to show a method to ecologically validate such mobility biomarkers.

## 1 Materials and methods

### 1.1 Participants

22 participants were recruited, 11 of whom had a clinical diagnosis of PD according to UK Brain Bank Criteria, with a Modified Hoehn and Yahr Scale score of 3 or less in the “OFF” medications state. This was a convenience sample size chosen to explore which parameters showed proof of concept and feasibility as potential markers of disease change in Parkinson's from in-home real-world data. The control cohort, included to show comparison to PD and to increase the safety and enjoyment of all in the study, consisted of spouses, close family members and friends of the PD participants; the participants took part in pairs (one person with PD, one control participant). Informed consent was gained from all participants. Full approvals (NHS Research Ethics Committee and Health Research Authority) were confirmed on 14th January 2020. The CONSORT diagram of recruitment is in the Multimedia Appendix.

### 1.2 Data collection

Each pair of participants stayed in an instrumented house for 5 days/4 nights continuously. Wall-mounted video cameras in the kitchen, hall, dining room and living recorded video for around 2 h a day at varying times according to participant preference with a resolution of 640/480 pixels and a frame rate of 30 frames/second (see study protocol [27]). During each pair's time in the house lived freely apart from when researchers visited (twice) to conduct clinical assessments. On one testing occasion, the PD participants undertook assessments twice: first in the practically defined “OFF” medications state then in the “ON” state having taken sufficient medication to achieve this.

The clinical assessments conducted by the clinician researcher included:

• MDS-UPDRS motor sub-scale
• A 20-m gait evaluation (in a 5-m corridor where the person turns 180° 3 times) performed at least 3 times each in the “ON” and “OFF” medications state for the participant with PD.

PD participants withheld their long-acting dopaminergic medication for 24 h and short-acting agents for 12 h before the practically-defined “OFF” data capture, including clinical assessments, on day 4. Participants with deep brain stimulators switched stimulation off 1 h prior to the “OFF” medication period clinical evaluation.

The “ON” medications video data was captured over around 6 h per participant pair split across 3 days, whereas the “OFF” medications data was from around 2 h of video on a single day (day 4). The timings were chosen for pragmatic reasons because of the participant discomfort burden of being “OFF” medications limiting such data captured to 1 day only, and the ethical approval to record 2 h of video each day during the study. The numbers of turns in each medication state are shown in Table 3.

### 1.3 Annotations

The videos were watched post-hoc by medical doctors who had undertaken training in the MDS-UPDRS rating score and had an interest in movement disorders; various aspects of the participants’ movement were identified and quantified, producing “annotations” (a set of parameter outcomes for each episode of turning of gait) [28]. A widely-available software called ELAN was used to watch up to 4 simultaneously-captured video files at a time. A pre-prepared annotation template was used by both clinician raters, with controlled vocabularies in drop-down menus to reduce the variability in the annotations created. The parameters annotated were: turning angle estimation (90°–360° in 45° increments), number of turning steps (integers from 1 to 18), duration of turn (seconds:milliseconds), type of turn (pivot turn, step turn).

A turning episode was defined as.

• Starting from the initiation of rotation of the pelvis, ending in completion of movement
• Not a turn taken in a walking arc (e.g. walking around a table)
• Clearly visible from the video

Where the participants’ feet were visible in the video frame, the number of turning steps was counted. In terms of type of turn: a pivot turn was classified as a turn in one or both feet swivel in place to achieve the turning movement; a step turn was classified as a turn achieved by three steps or more without pivoting.

### 1.4 Inter-rater agreement

Two clinicians annotated 50% of the turns each. Around 50% of the total number of annotations were cross-checked (randomly selecting 6 pairs from 11) by both clinician annotators, blinding the cross-checking clinician to the turning annotations produced by the other. Cohen's Kappa [29] statistic was calculated to evaluate inter-rater reliability. Any discrepancies were recorded, discussed, and resolved by the clinician raters, and with a final review by a movement disorders specialist.

### 1.5 Statistical approach

To investigate the correlations between the MDS-UPDRS III total score and the turning parameters, Spearman's rank correlation coefficients were used. To evaluate mean group differences (between PD and control; PD “ON” and “OFF” medication; free-living and clinical assessment turns), Wilcoxon rank-sum [30] tests were utilised. Because most turns taken during the clinical assessments were over a 180° angle (as shown in Table 1), only the 180° turns from both sets were used for statistical analysis comparing clinical assessment with free-living turns.

**Table 1:** Participant demographics.

Participant ID	Age (years)	Gender	Years since diagnosis of PD	H&Y Score in “OFF” state	LEDD (mg)/DBS	MDS-UPDRS motor sub-scale score “ON” medications/control	MDS-UPDRS motor sub-scale score “OFF” medications	TUGT time “ON” medications (secs)/control	TUGT time “OFF” medications (secs)
**PD participants**									
PD1	74	Female	5	3	475	17	21	9.7	11.7
PD2	60	Male	18	3	665	19	36	9.6	23.5
PD3	57	Male	17	2.5	666/DBS	20	35	6.7	9.7
PD4	57	Male	11	3	760	28	40	7.4	8.0
PD5	68	Female	19	3	1153/DBS	28	87	8.2	9.0
PD6	46	Female	0.5	1	75	10	14	5.2	5.6
PD7	72	Male	1	1.5	75	6	13	9.5	9.8
PD8	71	Male	2	2.5	400	19	23	8.0	9.5
PD9	62	Male	3	1	315	14	17	7.6	6.9
PD10	55	Male	6	2	410	32	36	5.0	5.8
PD11	59	Male	11	2	1216/DBS	17	47	11.1	8.7
**Control participants**									
1	76	Male				6		9.5	
2	54	Female				2		8.2	
3	65	Male				2		7.6	
4	58	Female				5		7.4	
5	23	Male				1		5.5	
6	72	Female				5		6.4	
7	71	Female				1		7.9	
8	60	Female				3		8	
9	57	Female				1		5	
10	58	Female				4		4.8	
11	57	Female				0		5.2	

LEDD = Levodopa equivalent daily dose (milligrams); DBS = deep brain stimulation; ID = identification; PD = Parkinson's disease; H&Y = Hoehn and Yahr; MDS-UPDRS = Movement Disorders Society sponsored revision of the Unified Parkinson's Disease Rating Scale; TUGT = Timed Up and Go Test.

## 2 Results

The demographics and clinical characteristics for the 22 participants are shown in Table 1. The mean ages were 61.9 (PD) and 59.2 (controls) years.

### 2.1 Numbers of turns captured

In total, 85.0 h of video footage was captured and annotated. Table 2 shows that from this data 3869 turns were observed in total and that over half of these were turns over a 90° angle. There were 22.7 turns per hour per person on average.

**Table 2:** Numbers of turns of different angles captured across the participant cohorts, during clinical assessments and free-living.

Turning angle (degrees)	Parkinson's disease			Control				%
	Free-Living (n)	Clin Ax (n)	Total (n)	Free-Living (n)	Clin Ax (n)	Total (n)	Total turns at different angles (n)	
**90**	1135	43	1178	1065	32	1097	**2275**	58
**135**	285	4	289	209	6	215	**504**	13
**180**	248	317	565	274	229	503	**1068**	28
**225**	3	0	3	9	0	9	**12**	0.3
**270**	4	0	4	0	0	0	**4**	0.1
**315**	0	0	0	1	0	1	**1**	0.03
**360**	0	0	0	5	0	5	**5**	0.1
Totals (n)	1675	364	**2039**	1563	267	**1830**	**3869**	

Clin Ax = Clinical Assessments.

**Table 3:** Table comparing mean numbers of turning steps and duration of turn between “ON” and “OFF” medication states in the participants with PD, in order of participants’ disease duration.

Participant ID number	Number of turning steps *Mean (confidence values)*		Duration of turn *Mean (confidence values)*	
	“ON” medications	“OFF” medications	“ON” medications	“OFF” medications
PD 6 *DD 0.5 years*	2.07 [1.93, 2.22] N = 174	2.08 [1.87, 2.30] N = 61	1.59 [1.50, 1.68] N = 174	1.49 [1.41, 1.57] N = 61
PD 7 *DD 1 year*	2.45 [2.28, 2.63] N = 110	2.80 [2.49, 3.10]* ↑ N = 44	1.83 [1.69, 1.97] N = 110	1.99 [1.81, 2.17]* ↑ N = 44
PD 8 *DD 2 years*	3.54 [3.22, 3.87] N = 143	2.92 [2.26, 3.58]* N = 24	2.53 [2.37, 2.69] N = 155	1.85 [1.70, 2.01]*** N = 25
PD 9 *DD 3 years*	2.95 [2.81, 3.09] N = 148	2.88 [2.43, 3.32] N = 32	2.28 [2.16, 2.40] N = 148	1.99 [1.64, 2.35]** N = 32
PD 1 *DD 5 years*	2.24 [2.10, 2.38] N = 166	2.35 [2.08, 2.62] N = 72	2.18 [2.07, 2.29] N = 166	2.00 [1.83, 2.16] N = 72
PD 10 *DD 6 years*	3.50 [3.04, 3.95] N = 91	2.81 [2.50, 3.11] N = 31	2.27 [2.07, 2.46] N = 101	2.15 [1.90, 2.40] N = 31
PD 4 *DD 11 years*	5.10 [4.64, 5.57] N = 97	6.40 [5.39, 7.41]* ↑ N = 22	2.47 [2.27, 2.68] N = 105	4.32 [3.24, 5.41]*** ↑ N = 25
PD 11 *DD 11 years*	2.75 [2.52, 2.98] N = 72	4.14 [3.56, 4.72]*** ↑ N = 21	2.18 [2.03, 2.32] N = 72	3.15 [2.48, 3.81]** ↑ N = 21
PD 3 *DD 17 years*	3.19 [2.77, 3.61] N = 119	4.55 [3.46, 5.64]** ↑ N = 24	1.84 [1.71, 1.96] N = 133	2.09 [1.63, 2.55] N = 31
PD 2 *DD 18 years*	1.88 [1.78, 1.99] N = 301	5.75 [4.67, 6.83]*** ↑ N = 28	1.30 [1.23, 1.36] N = 301	1.91 [1.65, 2.18]*** ↑ N = 28
PD 5 *DD 19 years*	3.20 [2.98, 3.42] N = 178	6.35 [5.39, 7.30]*** ↑ N = 23	2.86 [2.62, 3.11] N = 178	4.27 [3.12, 5.43]** ↑ N = 26

*ID = identification; DD = PD disease duration; N = number of turning episodes;* *p < 0.05, **p < 0.01, ***P < 0.001.

### 2.2 Inter-rater agreement

The two clinician raters had an almost perfect [31] inter-rater agreement for turning angle (Cohen's kappa = 0.96) and number of turning steps (Cohen's kappa = 0.97) annotations.

### 2.3 “ON” and “OFF” medication state in the participant with PD

Table 3 shows that 6 out of 11 participants with PD had an increase in mean number of turning steps when “OFF” medications compared to when “ON” medications, and 5 out of 11 showed an increase in mean turn duration in the same comparison (looking at all turning angles, from free-living data only)

One participant (PD 8) showed fewer turning steps and a shorter mean turning duration when “ON” medications compared to “OFF” medications, and PD 9 also had a shorter mean duration of turn when “ON” medications compared to “OFF”.

Two PD participants (PD 2 and PD 5, the 2 subjects with the longest duration of disease) show significantly more step turns taken when they are in the “OFF” medication state compared to when they were “ON” medications: PD 2% of step turns from total turns “ON” = 19%, “OFF” = 93%, p-value < 0.001; PD 5% of step turns from total turns “ON” = 31%, “OFF” = 65%, p-value < 0.001.

### 2.4 PD vs. control

The PD cohort (n = 11) had a significantly higher average number of turning steps (mean = 2.95, 95% confidence interval [2.87, 3.03], SD = 1.88) than the control cohort (n = 11, m = 2.36 [2.29, 2.43], SD = 1.56, W = 1347763, p < 0.001) across all turns. The PD cohort also had a longer mean duration of turn in seconds (m = 2.06 [2.02, 2.11], SD = 1.05) than the control participant cohort (m = 1.65 [1.62, 1.68], SD = 0.59, W = 1438171, p < 0.001).

### 2.5 Correlations

**Fig. 1:**
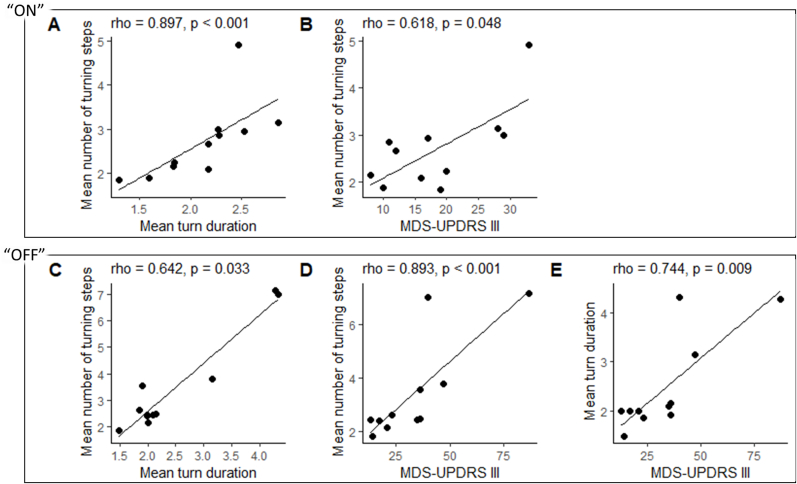
Illustration of the correlations between the turning parameters and the MDS-UPDRS III outcome scores, visualized as scatter plots, first “ON” then “OFF” medications.

Table 4 shows that there is a strong correlation between the mean number of turning steps and mean turn duration in the PD cohort when the participants are free-living “ON” medications (rho = 0.897). It also shows a significant positive correlation between the MDS-UPDRS motor sub-score (III) and the mean number of turning steps in free-living when the participant is “ON” (rho = 0.618). There is a significant correlation between mean number of turning steps and turn duration in the PD participants while “OFF” medication in free-living (rho = 0.642). There is a strong positive correlation between the MDS-UPDRS III motor sub-score and both the mean number of turning steps (rho = 0.893) and mean turn duration (rho = 0.744) “OFF” medications. False discovery rate adjustment was performed for dependent tests using the Benjamini-Hochberg procedure [32]. Where p-values have been corrected, they are reported alongside the number of dependent tests. The positive correlations are visualized in scatter plots in Fig. 1.

**Table 4:** Table showing correlations between “ON” and “OFF” medication turning parameters from the PD participant cohort in free-living and clinical rating outcomes (all turning angles).

			Free-living			
Medication status	Variable		Mean no turning steps		Mean turn duration	
“ON”	Mean turn duration	Spearman's rho	0.897	*** a	–	
		p-value	<.001		–	
		B–H p-value	0.004 (n = 4)			
	MDS-UPDRS III	Spearman's rho	**0.618**	*	0.469	
		p-value	0.048		0.145	
“OFF”	Mean turn duration	Spearman's rho	**0.642**	*	–	
		p-value	0.033		–	
	MDS-UPDRS III	Spearman's rho	**0.893**	*** a	**0.744**	** a
		p-value	<.001		0.009	
		B–H p-value	0.004 (n = 4)		0.027 (n = 3)	

*p < 0.05, **p < 0.01, ***P < 0.001.

a Survived false discovery rate adjustment using Benjamini-Hochberg (B–H) procedure.

### 2.6 Clinical assessments vs. free-living

Table 5 shows the comparison between free-living and clinical assessment turning over 180°. The PD participant cohort took significantly more steps to turn during clinical assessments (mean = 4.75 [4.47, 5.04]) compared to free-living (mean = 3.58 [3.36, 3.81], n = 11, W = 27304, p < 0.001). The increase in mean number of turning steps was also seen with statistical significance in six individual participants with PD; six PD participants also had a shorter mean turn duration in clinical assessments compared to free-living.

**Table 5:** Table showing turning differences between clinical assessment and free-living for turns of 180° for all participants.

Participant ID number	Number of turning steps *Mean (confidence values)*		Duration of turn (seconds) *Mean (confidence values)*	
	Free-living	Clinical assessment	Free-living	Clinical assessment
PD 1	2.98 [2.71, 3.24] N = 203	3.50 [3.17, 3.83]* ↑ N = 35	2.57 [2.35, 2.78] N = 203	2.26 [2.06, 2.45] N = 35
PD 2	2.77 [2.42, 3.12] N = 304	5.86 [4.48, 7.25]*** ↑ N = 25	1.78 [1.59, 1.98] N = 304	4.38 [2.79, 5.96] N = 25
PD 3	2.38 [2.13, 2.63] N = 108	6.02 [5.04, 7.00]*** ↑ N = 35	1.97 [1.73, 2.21] N = 108	1.71 [1.57, 1.84] N = 56
PD 4	6.30 [5.40, 7.20] N = 99	6.17 [5.02, 7.33] N = 20	3.04 [2.54, 3.54] N = 103	2.09 [1.88, 2.31]** ↓ N = 27
PD 5	4.41 [3.61, 5.22] N = 156	3.88 [3.36, 4.39] N = 45	3.37 [2.76, 3.99] N = 158	1.94 [1.76, 2.12]*** ↓ N = 46
PD 6	2.95 [2.57, 3.33] N = 203	3.48 [3.20, 3.77]* ↑ N = 32	2.11 [1.77, 2.44] N = 203	1.73 [1.48, 1.98]* ↓ N = 32
PD 7	3.08 [2.66, 3.49] N = 116	3.73 [3.44, 4.01]* ↑ N = 38	2.22 [1.78, 2.66] N = 116	2.05 [1.88, 2.22] N = 38
PD 8	3.29 [2.84, 3.74] N = 148	6.41 [5.20, 7.63]*** ↑ N = 19	2.81 [2.38, 3.24] N = 148	1.92 [1.75, 2.09]*** ↓ N = 32
PD 9	3.94 [3.51, 4.37] N = 152	3.87 [3.37, 4.37] N = 28	2.97 [2.63, 3.32] N = 152	2.15 [1.83, 2.46]*** ↓ N = 28
PD 10	4.31 [2.86, 5.75] N = 95	4.77 [3.84, 5.70] N = 27	2.46 [1.84, 3.08] N = 99	1.71 [1.55, 1.87]* ↓ N = 33
PD 11	4.00 [2.04, 5.96] N = 81	4.27 [3.68, 4.87] N = 12	2.62 [1.13, 4.11] N = 81	2.37 [2.03, 2.71] N = 12
C 1	2.67 [1.36, 3.97] N = 80	3.19 [2.90, 3.48] N = 24	2.65 [1.98, 3.32] N = 80	1.82 [1.63, 2.00]* ↓ N = 24
C 2	3.33 [1.67, 5.00] N = 222	5.92 [4.55, 7.30]* ↑ N = 12	1.50 [1.31, 1.70] N = 222	1.94 [1.76, 2.12]* ↑ N = 12
C 3	2.83 [2.47, 3.20] N = 36	1.71 [1.35, 2.08]** ↓ N = 15	2.23 [1.96, 2.50] N = 38	1.16 [1.05, 1.27]** ↓ N = 26
C 4	3.20 [2.15, 4.25] N = 86	4.83 [3.30, 6.36]* ↑ N = 28	1.99 [1.62, 2.35] N = 89	1.82 [1.54, 2.11] N = 34
C 5	3.17 [2.79, 3.56] N = 118	4.96 [3.85, 6.08]* ↑ N = 29	2.57 [2.26, 2.89] N = 118	1.55 [1.39, 1.71]*** ↓ N = 36
C 6	2.32 [2.05, 2.58] N = 145	2.50 [2.09, 2.91] N = 20	1.84 [1.57, 2.10] N = 145	1.70 [1.44, 1.96] N = 20
C 7	2.26 [2.05, 2.47] N = 272	2.48 [2.22, 2.73] N = 23	1.68 [1.53, 1.82] N = 273	1.31 [1.12, 1.50]* ↓ N = 23
C 8	3.14 [2.74, 3.55] N = 85	6.19 [4.90, 7.48]** ↑ N = 17	2.24 [1.97, 2.51] N = 85	1.77 [1.62, 1.93]** ↓ N = 28
C 9	2.64 [2.49, 2.79] N = 215	3.33 [2.92, 3.75]** ↑ N = 15	2.20 [2.02, 2.39] N = 215	1.49 [1.30, 1.68]*** ↓ N = 15
C 10	2.85 [2.64, 3.06] N = 164	5.77 [4.41, 7.12] N = 17	1.99 [1.81, 2.17] N = 165	1.40 [1.27, 1.54]*** ↓ N = 30
C 11	2.78 [2.44, 3.12] N = 133	2.82 [2.48, 3.17] N = 19	2.13 [1.92, 2.34] N = 133	1.50 [1.31, 1.69]*** ↓ N = 19

*P < 0.05, **P < 0.01, ***P < 0.001.

The control participant cohort also took more turning steps on average to make a 180° turn when undertaking clinical assessment (mean = 4.34 [3.95, 4.72]) compared to free-living (mean = 2.72 [2.59, 2.85], W = 21228, p < 0.001). 5 control individuals showed significantly increased mean number of turning steps while 1 (C3) conversely took fewer steps during clinical assessments. The control cohort overall also had shorter mean turn durations when they were being observed during clinical assessment compared to free-living (p < 0.001) and 8 control participants supported this trend at an individual level.

## 3 Discussion

This is the first work to show that it is feasible to quantify reliable parameters of free-living turns (number of turns, number of turning steps, duration of turn, turning angle) from wall-mounted cameras in a home-like setting in PD. We have shown that these turning parameters can differentiate between PD and control participants and they show significant individual-level differences between the “ON” and “OFF” medication state in several PD participants. Additionally, there are strong correlations between the turning parameters and the MDS-UPDRS III scores in the PD cohort, particularly marked when “OFF” medication (the first time such a comparison has been done from in-home free-living data). An unexpected but potentially impactful finding is the presence and scale of change in turning behaviors when doing observed clinical assessments compared to “unobserved” (for these purposes, meaning no clinician physically present) free-living in this real-world setting.

In this study, we have demonstrated that people make >20 turns on average per person per hour while free-living. Over a longer time monitoring in-home turning, the resulting dataset will potentially be rich and informative about naturalistic mobility outcomes. This is important as indoor turning outcomes can complement straight-ahead gait parameters [21,24], which are better measured outdoors (due to space restraints in most homes), to achieve a 24-h view of free-living mobility. 58% of the turns seen are taken over 90°, which is important since the majority of observed turns in clinic are over 180° - therefore we could be missing valuable gait-related information in clinic.

Five participants with PD showed an increase in both the average number of turning steps and average duration of turn “OFF” medications, in comparison with their “ON” medication turns (one further PD participant showed this trend of increasing only number of turning steps “OFF” medications). Such changes show the promise of these turning parameters to detect dopamine-related gait fluctuations in PD. Two PD participants (PD 8 and PD 9) showed the opposite trends: their duration of turn (both participants) and number of turning steps (only PD 8) reduced “OFF” medications. There are several potential explanations for these unpredicted findings, including the fact that both participants noted significant issues with orthostatic hypotension in the “ON” state which may have led to them turning slower “ON” medications. PD 8 also reported excessive daytime sleepiness (affecting mobility) in the morning, associated with sleep disturbance, and had slept worse according to their study diary in the nights before their “ON” medication data was collected than before their “OFF” medication period. Two participants with the greatest duration of PD took significantly more step turns while “OFF” medications compared to their “ON” medication state, in a reflection of how step turns appear later in the disease process and therefore this difference was ‘unmasked’ only in these two people with dopaminergic medications were withheld. The difference between individuals, and how medication-responsive their mobility outcomes were, highlights the compelling argument to stratify patients into their PD phenotypes in clinical trials to better appreciate the individual significance of mobility-related outcomes [33].

This work found, at a cohort level, that the PD participants take more turning steps and have longer durations of turn compared to the control participants (although a limitation is that the male/female ratios differ between the two groups). This supports previous works evaluating how turning differs between PD and control in the clinic [22]. However, to truly understand how free-living turning differs between PD and non-PD participants, larger cohorts are needed to reduce the impact of potential confounders (e.g. age and physical activity level). The parameters in this study are a subset of the potential turning outcomes which could be evaluated; they are particularly feasible to accurately rate by a human watching video, hence why they are reported on in this study. Future work could look at other aspects of turning such as gait stability in turning.

It is well-understood that (straight-ahead) gait parameters differ between the laboratory and home settings in PD [14,15]. However, this study adds information to this understanding in two ways: firstly, by showing marked differences in gait outcomes when *all* observations were made in a home-like “real-world” (not laboratory) setting, and secondly by looking specifically at changes in turning of gait as opposed to straight-ahead gait episodes. Differences seen between turning parameters in free-living compared to clinical assessments in this study may have occurred for a number of reasons: the Hawthorne effect of clinician and video recording; an increasing familiarity with tasks repeated several times; the single-task nature of the clinical assessment compared to potential dual-tasking in free-living (e.g. carrying a book and turning). Additionally, a physical setting can change how someone mobilizes [34]: the clinical assessments were undertaken in a relatively narrow hallway, whereas the free-living data was taken from any of the more open downstairs communal rooms or the hallway. Work has been done by other researchers to replicate naturalistic conditions in the laboratory in gait evaluation [35], but further work is needed to control for external influences on gait parameters in design of clinical trials. For now, this work raises awareness that turn duration and number of turning steps may not be representative of how someone turns at home.

The past few years have seen advances in how wearable sensors can automatically measure other aspects of gait and functioning including freezing of gait [36] and falls risk [37]. Mobility quality parameters, such as turn angle, can also be automatically quantified by wearable sensors [38], but real-world validation remains challenging, and few studies have shown technical validity of digital outcomes (often limited to laboratory or home-like environments) [18,23,39]. Beyond turning parameters, other gait parameters including those related to straight-ahead gait pace (including step velocity and length), variability, rhythm and asymmetry show promise in free-living as potential biomarkers in PD [21].

Importantly, the knowledge that the control participant cohort also altered the way they turned during clinical assessments has broader implications for other research groups looking at mobility. The impact of how the Hawthorne effect and other aspects of the clinical assessment, including the setting, changes turning outcomes should also be explored in other disease and non-disease groups.

### 3.1 Study strengths, limitations and future directions

This was a pilot study which had, as a particular strength, the use of multiple wall-mounted cameras collecting video recording unscripted and unobserved free-living behavior from people with PD in a home-like setting over multiple days. To the authors’ knowledge, this is the first such work to do this. A further strength was in the amount of data captured, 85 h in total, meaning that the number of turns available for analysis was almost 4000. The turning annotations had almost perfect inter-rater agreement, resulting in a high-quality dataset which helps real-world PD symptom understanding.

We felt that wall-mounted cameras achieved a good visualization of each room, were minimally invasive to the study participants (although appreciating that in-home cameras may not be acceptable to all people with PD, the opinion of these participants was largely very positive [25]) and obviated the need for researchers to be present.

Although this pilot recruited participants with a wide variety of disease severities, the small sample size means that the results are not currently generalizable to the wider population of people with PD. A knowledge gap also remains around whether the same correlations would be seen between turning parameters and the clinical rating scale outcomes if only early-stage PD participants were studied. We hypothesize that larger sample sizes would be needed potentially over longer periods of time but that turning behaviors would correlate with disease severity outcomes.

As discussed above, the physical setting could be seen both as a study strength (a naturalistic setting as opposed to a laboratory environment) and a study limitation (relatively narrow hallway; unfamiliar setting for the participants which may have altered how they mobilized). Future work is needed in peoples’ own homes with this scalable equipment, potentially capturing both free-living and structured assessments over longer periods of time, to get a true picture of how to characterize ecologically valid gait parameters for use as a marker of progression in disease-modifying therapeutic investigations.

The turning of gait parameters quantified were designed to be reproducible by clinicians watching similar videos in the future, including measures of quantity and quality of turning [20], but this approach to provide a ground truth with which to inform other sensors’ data is burdensome in time and effort. Further work needs to be done to automate turning detection and quantification rather than using human-created annotations, even if these are a vital first component to achieving ecological validation of mobility parameters in the real world.

## 4 Conclusions

This work has shown that turning episodes occur frequently in free-living and can be captured and annotated manually using video data with excellent inter-rater agreement. It has demonstrated that turning parameters can differentiate the “ON” dopaminergic medication state from the “OFF” state in PD in some patients, and between PD and control participant cohorts. The turning parameters from 5-days of real-world data correlated with the gold-standard clinical outcome of disease severity in PD, demonstrating their potential for use as markers of disease progression. Importantly, even within this same home-like setting, many participants turned differently when undertaking clinical assessments compared to when they mobilized in free-living. Further work is needed to understand the impact of the study setting and Hawthorne effect on how someone with PD turns. In aggregate, we feel the above encourages future scalability work including, crucially, developing ecologically validated automatic approaches to detect turning of gait and quantify turning parameters in-home.

## Funding

This work was supported by the SPHERE Next Steps Project funded by the 10.13039/501100000266UK Engineering and Physical Sciences Research Council (EPSRC), [Grant EP/R005273/1]; and the Elizabeth Blackwell Institute for Health Research, University of Bristol and the 10.13039/100010269Wellcome Trust Institutional Strategic Support Fund [grant code: 204813/Z/16/Z]; and by Cure Parkinson's [grant code AW021]; and by 10.13039/501100015725IXICO [grant code R101507-101]. Dr Jonathan de Pass and Mrs Georgina de Pass made a charitable donation to the University of Bristol through the Development and Alumni Relations Office to support research into Parkinson's Disease. The funding pays for the salary of CM, the PhD student and Clinical Research Fellow, and she reports to the donors on her progress.

## Data statement

Data is not available at this point due to confidentiality of participants, but we hope to make the data available in due course as openly as our participant consent allows through the University of Bristol Data Repository Service.

## Ethical compliance statement

Full approval from NHS Wales Research Ethics Committee 6 was granted on 17th of December 2019, and Health Research Authority and 10.13039/100012068Health and Care Research Wales approval confirmed on 14th of January 2020; the research was carried out in accord with the Helsinki Declaration of 1975.

Written informed consent was gained from all study participants.

## Figures and Tables

**Figure:**
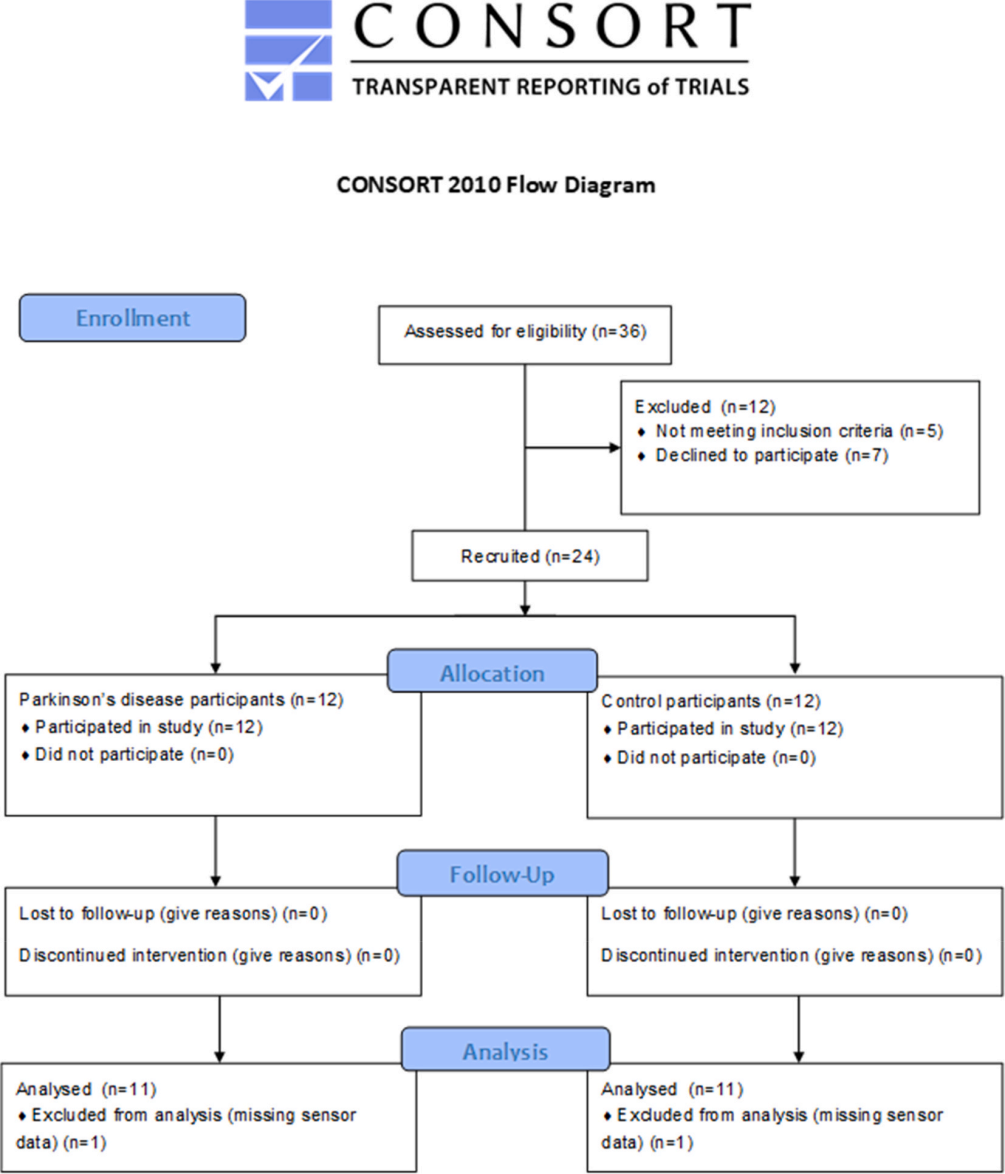


## Appendix

CONSORT 2010 Flow Diagram for Study Recruitment.
